# Optimization of magnetic nano-iron production by *Aspergillus flavipes* MN956655.1 using response surface methodology and evaluation of their dye decolorizing and antifungal activities

**DOI:** 10.1038/s41598-022-25339-3

**Published:** 2022-12-06

**Authors:** Nashwa H. Abdullah

**Affiliations:** Botany and Microbiology Department, Faculty of Science, Helwan University, Helwan, Egypt

**Keywords:** Biotechnology, Microbiology

## Abstract

Iron nanoparticles have been biosynthesized by a new *Aspergillus flavipes* isolate. Size of biosynthesized iron nanoparticles was in a range of 32.7 and 47.6 nm, their surface charge was − 33.5 ± 5.3 and they showed semihard ferromagnetic behavior. Salt concentration, volume of added culture filtrate and pH have been optimized using response surface methodology. A significant effect for the added culture filtrate and a mutual interaction between this factor and the pH has been detected. Model validation results showed 3.3% deviation from the statistically predicted values which reflects the accuracy of the employed model. Optimization process has increased the quantity of iron in the prepared samples and the amount of produced iron nanoparticles to a fourfold. The optimized conditions have stimulated the formation of nanoparticles in a tetrahedron shape rather than the truncated tetrahedron shape without affecting their size or surface charge. The biosynthesized iron nanoparticles have recorded a good decolorization activity for methylene blue. They showed 57 ± 4.3 decolorization percent after 6 h when tested with only 0.1 mg/ml concentration. Moreover, 50 ppm concentration has exerted a detectable antifungal activity against *Alternaria solani*. This study represents a new competitive green synthesis method for magnetic iron nanoparticle.

## Introduction

Nanotechnology is a branch of science that deals with the use of materials at nanoscale. This concept was firstly introduced in 1959 by Richard Feynman and the term “nanotechnology” was firstly used by a Japanese scientist called Norio Taniguchi in 1974^1,2^.

A nanoparticle can be defined as a minute particle with dimensions ranging between 1 and 100 nm (10^–9^ m). It can be considered as a turning point between bulk material and atomic-scale material^2,3^ Compared to their bulk materials, nanoparticles have unique and enhanced characters due to their higher surface to volume ratio^4^. As a result, they show higher reactivity, higher surface potential, tunable physical/chemical properties^1^. In addition, they may show extraordinary thermal, optical, and electrochemical properties which give them a great value in many application fields such as the industrial, agricultural, medical and environmental fields^3^.

Nanoparticles can be produced by two techniques “Top-down” approach and “Bottom-up” approach^2,4^. In case of “Top-down” approach, nanoparticles are produced from their bulk materials utilizing physical methods such as mechanical milling, laser ablation and sonication. These techniques are time-consuming and yield non-uniformly distributed particles. On the other hand, “Bottom-up” approach builds up the nanoparticles from their molecular base utilizing chemical and biological techniques^4^. Chemical strategies usually involve the use of harmful compounds and release some toxic byproducts making them unfavorable methods due to the resulted environmental issues^5^. In biological methods, biological components are utilized for synthesis process. This method is considered as a promising technique as it is safe, eco-friendly, and economically effective. Moreover, it yields well-defined sized nanoparticles^4,6^.

Amongst the metallic nanomaterials, iron nanoparticles have gained a great attention due to their low toxicity, good reactivity, and magnetic behavior. Such characteristics make them one of the best competitors in nano science. Indeed, iron nanoparticles have been efficiently used in medicine, agriculture, food, paint, textile, cosmetics, and wastewater treatment fields^4^. Magnetic behavior of iron nanoparticles added a special significance to them in certain applications such as magnetic, electrical and biomedical applications. This magnetic behavior has a great value in directed drug delivery, labeling, magnetic separation of biological materials^3^ in addition to the wastewater treatment.

Plants, bacteria and fungi have been utilized in green/bio synthesis of iron nanoparticles; however fungal mediated methods can be considered as the most efficient one as fungi are potent producers of bioactive compounds which play a role in the production process of nanoparticles as reductive agents^7^. Moreover, fungi are easy to be handled, show fast growth rate and its cultivation and maintenance methods are costly effective^6^. Despite that, little studies have been performed on myco-synthesis of zero valent iron nanoparticles.

Thus, the present study has focused on the use of fungi in green synthesis of magnetic iron nanoparticles. Moreover, this study has investigated how to optimize some parameters in the production process to enhance their productivity.

Optimization process is traditionally carried out by the one factor at time approach (OFAT)^8,9^. In this technique one factor is studied at a time while the other factors are kept constant^9^. Hence, this approach is a time-consuming where the factors must be optimized one by one. Moreover, such method ignores the mutual interaction which possibly occurs between the tested factors. Therefore, quality professionals prefer to use other statistically based methods such as design of experiment (DOE) methods^8,9^ which can estimate both the main effect and the interaction between the tested factors^9–12^. Response surface methodology is one of such statistically designs that generates a response surface map and estimates how to move the process to the optimum location^8^. Consequently, this method has been selected to perform the employed optimization study.

Finally, the effectiveness of the produced iron nanoparticles as dye decolorizing and antifungal agent has been investigated in a trial to evaluate their applied value.

## Methods

### Fungal isolate and its identification

The investigated fungus has been isolated from soil sediment of *Avicennia marina* (Forssk.) Vierh. growing on Red Sea coast at Ras Mohammad—South Sinai—Egypt (27°44′30″ N, 34°14′05″ E) using soil dilution plate method described by Waksman^13^ and Kumar et al.^14^. This isolate has been identified by both molecular and morphological methods. Genomic DNA was extracted from the fungal mat using cetyl trimethylammonium bromide (CTAB) method^15,16^. ITS4 primer was used for amplification of ribosomal internal transcribed spacer (ITS). PCR product was purified and sequenced at Macrogen Company—Seoul—South Korea. The obtained DNA sequence was identified utilizing Basic Local Alignment Search Tool (BLAST) available on the website of National Center of Biotechnology Information (NCBI). Phylogenetic analysis was performed using (NCBI) and MegAlign (DNA Star) software version 5.05. The molecular identification was confirmed by studying the growth, morphological and microscopical characters of the investigated isolate.

### Biosynthesis of iron nanoparticles

The ability of obtained fungal isolate to synthesis iron nanoparticles extracellularly has been investigated. Two fungal discs (0.8 cm diameter using cork borer) were inoculated to 250 ml conical flasks, containing 50 ml of MGYP broth medium (Malt extract 3.00, Yeast extract 3.00, Peptone 5.00, Glucose 10.00 g/l, pH = 6.2;^17^, and incubated at 25 °C for 14 day under static conditions. Then the grown mycelium was separated by filtration using Whatman filter paper no.1. The culture filtrate was taken and filtered using 0.45 µm sterile cellulose nitrate membrane filter (Sartorius; Germany) and used for iron nanoparticles production extracellularly as following; 10 ml of culture filtrate was inoculated in 90 ml FeSo_4_.7 H_2_O solution in a final salt concentration of 0.1 mM, pH = 5.7. The mixture was incubated at room temperature at 150 rpm for 2 h. Iron nanoparticles production was detected by observing color change of sample and measuring its UV–Vis spectrum in comparison to salt free control in the range of 200–800 nm (JASCO V630). The UV–Vis spectrum of control sample containing zero salt concentration was used as the basal line for detecting the zero valent iron nanoparticle peaks. This test was carried out in triplicate manner.

### Monitoring of iron nanoparticles biosynthesis over different time intervals

Behavior of biosynthesis of iron nanoparticles over time was monitored by incubating the reaction mixture for different time periods (30 min, 2 h, 6 h and 24 h) and measuring UV-Vis spectrum of samples in a range of 200–800 nm to track the appearance of iron nanoparticles beak.

### Characterization of biosynthesized iron nanoparticles

#### Dynamic light scattering (DLS) and transmission electron microscopy (TEM)

Size of synthesized iron nanoparticles was initially checked by Dynamic light scattering (DLS) technique using nano-zeta sizer (Malvern ZS-Nano, Scattering angle of 90). The dynamic light scattering technique is ideal for the measurement of the size of colloids and nanoparticles. The principle of that technique is that the fine particles and molecules are in constant Brownian motion, which its speed depends on their size. This speed can be monitored using the speckle pattern produced by illuminating the particles with a laser. The scattering intensity at a specific angle fluctuating with time is detected using a sensitive avalanche photodiode detector (APD). The intensity changes are analyzed with a digital autocorrelator which generates a correlation. This curve can be analyzed to give the size and the size distribution.

Additionally, the estimated size range was further confirmed by employing Transmission Electron Microscopy (JEM 2100 HRT, Japan, 200 kV).

#### Vibrating sample magnetometer (VSM)

Vibrating sample magnetometer was employed to evaluate the magnetic behavior of synthesized iron nanoparticles (iron NPs) at room temperature. Measurement was carried out with a magnetic field up to 20 kG. The vibrating sample magnetometer (VSM) is one of the most successful operation methods of a magnetometer. VSM measurements are unaffected with mass and size of sample.

#### Fourier-transform infrared (FT-IR) spectroscopy

Nature of compounds involved in the production and capping of synthesized iron nanoparticles have been checked by estimating the type of functional groups and bonds found in both the crude culture filtrate and biosynthesized iron nanoparticles. Spectra were detected using (Alpha II, Bruker, USA) at range of 4000–400 cm^−1^ and the measurements were recorded as the percent Transmission (% T).

### Optimization of some production parameters using response surface methodology

Levels of some vital factors that can affect the nano iron synthesis have been optimized employing response surface methodology method “using Box-Behnken design”. Starting salt concentration, volume of added culture filtrate and pH were the tested factors. These three factors have been monitored at three levels, high (+ 1), low (− 1), and intermediate (0) as shown in Table 1.

**Table 1:** Levels of factors selected for optimization study using Box-Behnken design.

Factor	Unit	Low level (− 1)	High level (+ 1)	Intermediate level (0)
Salt*^1^ concentration	mM	0.1	2	1.05
Filtrate*^2^ volume	ml	1	20	10.5
pH	–	4	10	7

*^1^FeSo_4_·7 H_2_O.

*^2^*Aspergillus flavipes* MN956655.1 Culture filtrate.

The experiment was performed in fifteen trials, Table 2, three of them are central points to avoid error. Size of obtained particles was measured by DLS technique using nano-zeta sizer (Malvern ZS-Nano, with a scattering angle of 90) and that considered as the main monitored response. Results were fitted with a second-order polynomial equation which its general form is:

$$ Y = \beta_{0} + \sum {\beta_{i} X_{i} } + \sum {\beta_{ij} X_{i} X_{j} } + \sum {\beta_{i} X_{i}^{2} } $$

**Table 2:** Matrix of Box-Behnken design employed for optimizing size of iron nanoparticles produced by *Aspergillus flavipes* (MN956655.1).

Trail no	Salt*^1^ Conc. (mM)	Filtrate*^2^ volume (ml)	pH	Response (Particle size nm)
1	2.000	1.00	7.00	434
2	0.100	10.50	10.00	377
3	0.100	10.50	4.00	396
4	1.050	1.00	10.00	281
5	0.100	1.00	7.00	432
6	2.000	10.50	10.00	320
7	1.050	10.50	7.00	176
8	1.050	20.00	10.00	270
9	1.050	10.50	7.00	59
10	2.000	10.50	4.00	480
11	1.050	1.00	4.00	491
12	2.000	20.00	7.00	179
13	1.050	10.50	7.00	176
14	1.050	20.00	4.00	49
15	0.100	20.00	7.00	131

*^1^FeSo_4_·7H_2_O.

*^2^*Aspergillus flavipes* MN956655.1 Culture filtrate.

*Y*: predicted response, β_0_: the intercept term, β_*i*_: the linear coefficient, β_*ij*_: the quadratic coefficient, β_*ii*_: the interaction coefficient, and *X*_*i*_*X*_*j*_: the independent variables^18^.

Statistical analysis was performed on obtained data using software package ‘Design Expert’ software (Version 7.0). Accuracy of polynomial model equation was evaluated by checking the values of *R*^*2*^ and adjusted *R*^2^. Three-dimensional response surface plots have been used to express the fitted polynomial equation. Optimizing the levels of tested factors was employed using the Design Expert’s numerical optimization tool in a trail to yield the best response (particle size). The goal of optimization process was to minimize the size of produced iron nanoparticles “minimize the response”.

### Experimental Validation

One of the predicted solutions that have been estimated by the numerical optimization tool for the best response results has been selected and tested experimentally. The percentage of deviation between the predicted and experimentally recorded values has been calculated to check and proof the accuracy of the obtained model.

### Assessment of production process after and before optimization study

Effectiveness of optimization process was evaluated by monitoring the amount and the characters of produced iron nano particles before and after applying the optimized conditions.

#### Zeta size and potential

Size and surface charge of synthesized iron nanoparticles have been measured before and after optimization process using nano-zeta sizer (Malvern ZS-Nano, Scattering angle of 90).

#### Scanning electron microscopy (SEM)

Shape and topography of synthesized iron nanoparticles before and after optimization study have been monitored using scanning electron microscopy (JEOL, JSM-6360LA, Japan).

#### Energy dispersive X ray (EDX) analysis

Percentage of iron in prepared samples before and after optimization process was checked using EDX along with Scanning Electron Microscopy (JEOL, JSM-6360LA, Japan Scanning Electron Microscope coupled with an Energy Dispersive X-ray spectrometer).

#### Produced amount

Quantity of produced iron nanoparticles under optimized and nonoptimized conditions was assessed by measuring the weight of dried nanoparticles. 100 ml of prepared nanoparticle solution was dried on oven at 60 °C for 72 h, then dry weight of produced nanoparticles was measured. The test was carried out in duplicated manner and a zero-salt solution “reaction mixture with zero salt concentration” has been used as a control.

### Effectiveness of the produced magnetic iron nanoparticles as dye decolorizing and Antifungal agent

#### Dye decolorization

Decolorization of methylene blue by the biosynthesized iron nanoparticles in presence of H_2_O_2_ has been tested. One milligram of iron nanoparticles was added to ten ml methylene blue solution (0.1 mg/l) and one ml of 10% H_2_O_2_. Decolorization of dye in absence of iron nanoparticles also in absence of both H_2_O_2_ and iron nanoparticles has also been evaluated. The reaction mixture was incubated at room temperature for 3 and 6 h. Absorbance of samples was measured at 665 nm to track methylene blue decolorization^19^. Test was performed in duplicated manner and percent of decolorization was calculated as following:

$$ \% \;{\text{Decolorization}} = {\text{A}}_{0} - {\text{A}}_{{\text{t}}} /{\text{A}}_{0} \times 100 $$

A_0_ = initial absorbance (at zero time), A_t_ = absorbance after each time interval^19,20^.

#### Antifungal activity

Antifungal activity of the biosynthesized iron nanoparticles against four fungal plant pathogens (*Fusarium solani*, *Fusarium semitectum*, *Alternaria solani* and *Sclerotium rolfsii*) has been estimated in a concentration of 5 and 50 ppm. The test was performed using percent inhibition of mycelial growth method (PIMG)^21,22^. Iron nanoparticles have been added to warm Czapek-Dox’s agar medium (Sucrose 30.00, NaNO_3_ 3.00, KH_2_PO_4_ 1.00, MgSO_4_ 0.50, KCl 0.50, FeSO_4_ 0.01, Agar 20 g/l, pH = 7.0;^23^), with the tested concentrations, mixed well and poured on sterilized petri dishes. After solidification, 7-day old fungal plugs (0.8 cm) were inoculated on the plates and incubated at 25 °C for 5 days. The test was carried out in duplicated manner and Czapek-Dox’s agar plates with zero nanoparticles concentration were used as control. The result was recorded by measuring the growth diameter and calculating of PIMG as following:

$$ {\text{PIMG}} = {{{\text{G}}_{{\text{c}}} - {\text{G}}_{{\text{t}}} } \mathord{\left/ {\vphantom {{{\text{G}}_{{\text{c}}} - {\text{G}}_{{\text{t}}} } {{\text{G}}_{{\text{c}}} \times 100}}} \right. \kern-\nulldelimiterspace} {{\text{G}}_{{\text{c}}} \times 100}} $$

G_c_: Growth diameter in control plate. G_t_: Growth diameter in plates inoculated with nanoparticle.

### Statistical analysis

The comparisons between two groups were performed by two groups *t*-Test analysis at 95% confidence interval using Sigma plot statistical package version 12.5. Standard deviation for the mean values was estimated. Statistical analysis for DOE optimization study was carried out by ‘Design Expert’ software package Version 7.0.

## Results

### Identification of fungal isolate

**Figure 1:**
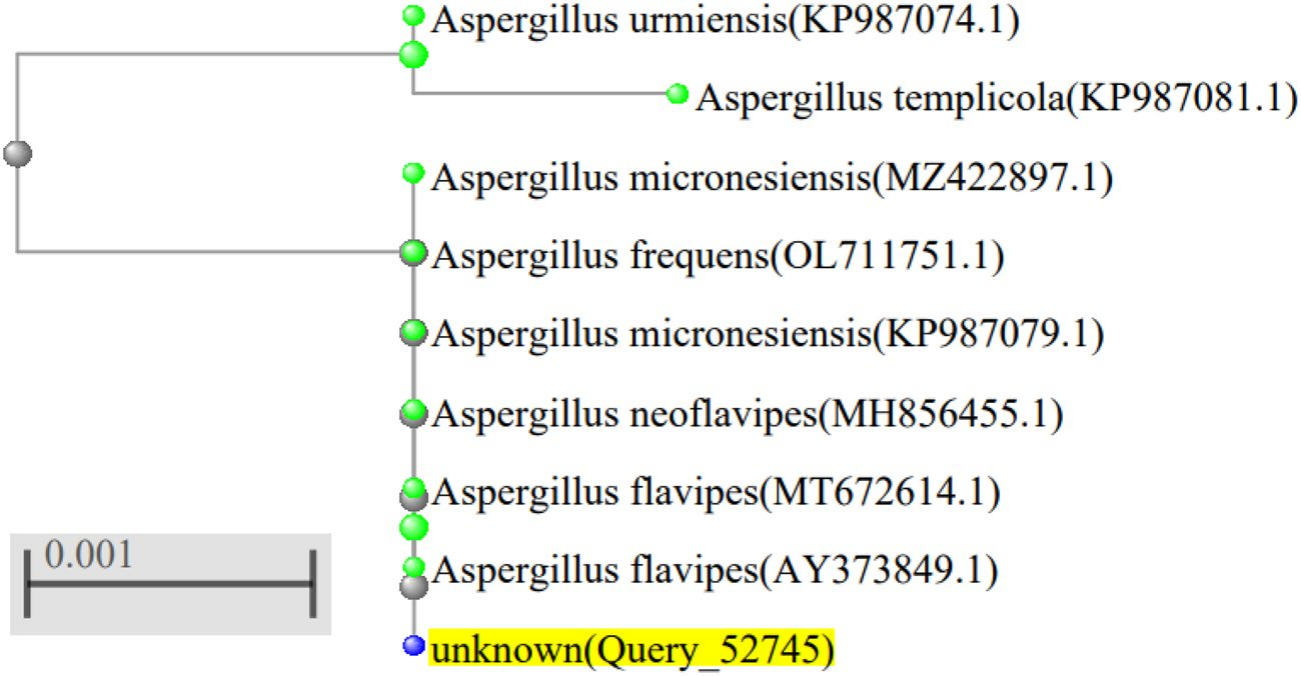
Phylogenetic tree analysis of obtained ITS4 sequence (NCBI); the analyzed sequence is highlighted.

**Figure 2:**
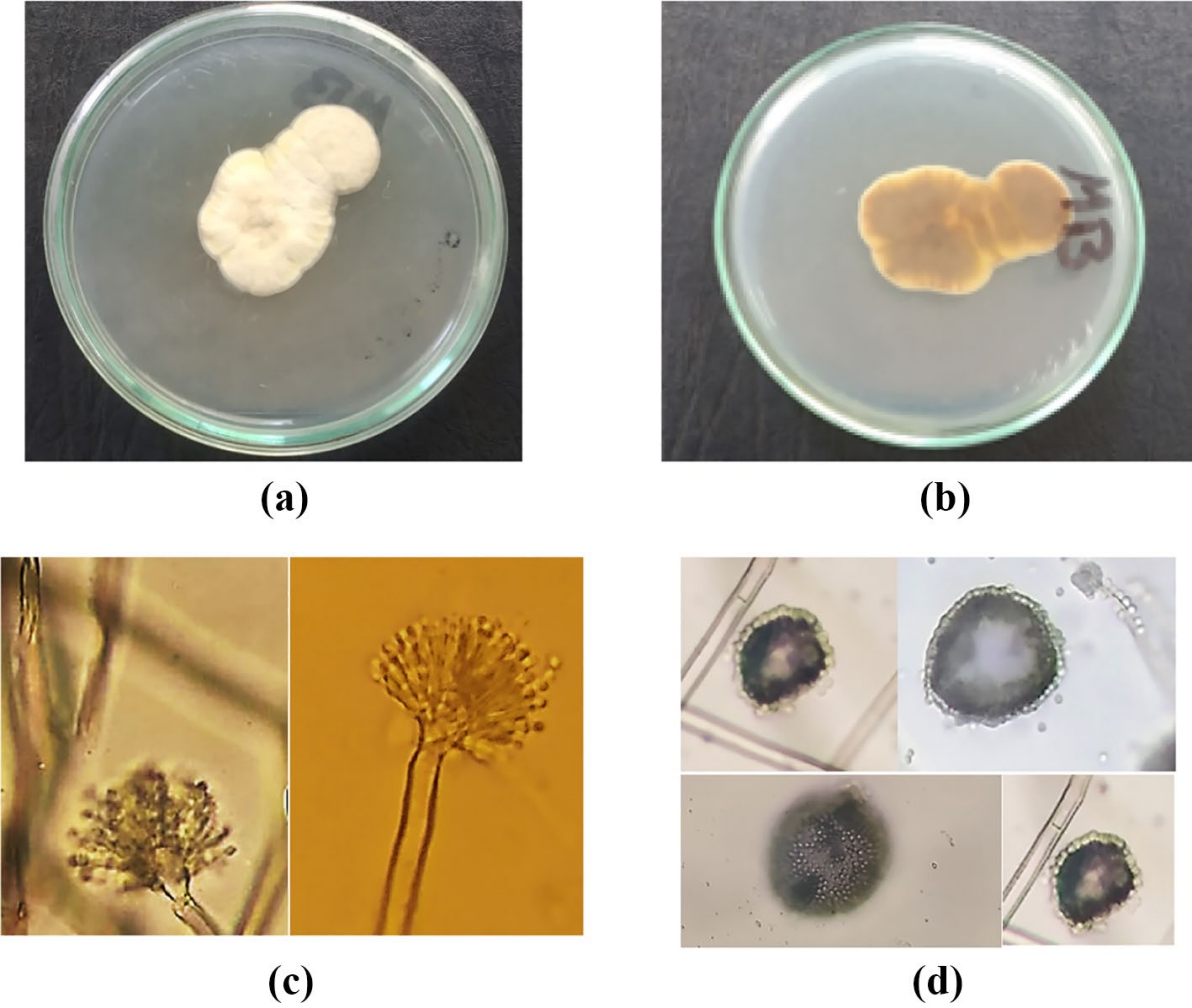
*Aspergillus flavipes* MN956655. (**a**); colony on Czapek’s Dox medium, (**b**); reverse of plate, (**c**); conidial head, (**d**); Hülle cells.

Sequencing of ITS4 region for the obtained isolate, revealed that; this isolate shows 99.45% similarity with different strains belonging to the following fungal species; *Aspergillus flavipes*, *Aspergillus micronesiensis*, *Aspergillus neoflavipes*. The phylogenetic tree analysis, Fig. 1, showed that; this isolate is found in a common joining with *Aspergillus flavipes* clusters, suggesting a new strain of *Aspergillus flavipes*. This identification was confirmed by checking the growth, macroscopic and microscopic characters of the investigated isolate. The investigated isolate on Czapek’s Dox media gives white to buff-yellowish colonies with off-white spores and reverse with brown shades producing no soluble pigments. These characters agree with characters described for *A. flavipes*^24–26^. On Malt Extract Agar (MEA) medium it gives similar characters. It showed no growth at 40 °C as described by Sklenář et al.^27^. Microscopical examination showed that, it forms spathulate conidial head. Swollen Hülle cells also have been observed and discrete masses of Hülle cells has been detected which agrees with microscopical features described for *A. flavipes*^25,28,29^, Fig. 2. According to the previous morphological characters the identification of the investigated isolate as *A. flavipes* has been confirmed. The previous growth and morphological description excludes the identification probabilities of *Aspergillus neoflavipes* and *Aspergillus micronesiensis* (*A. frequens*; *A. micronesiensis* synonymous) as *Aspergillus neoflavipes* shows bright yellow colonies on MEA^28,30^ and able to grow at 40 °C^27^, on the other hand, *Aspergillus micronesiensis* forms globose radiating conidial head and its colonies give soluble orange brown pigment^29^ and these characters do not match with the morphological and growth character of the investigated isolate. Thus, the obtained sequence was submitted in GenBank as *Aspergillus flavipes* isolate RMMB and released in GenBank under the accession number of MN956655.1.

### Biosynthesis of iron nanoparticles

**Figure 3:**
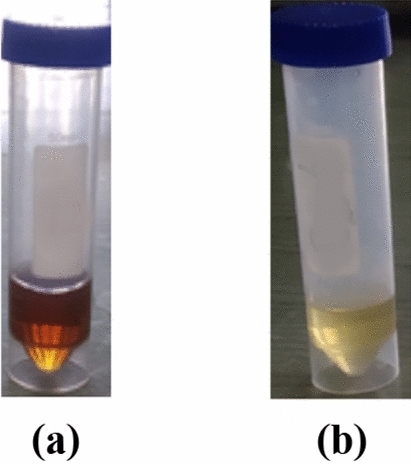
(**a**) Color change due to iron nano particles production, (**b**) control with zero salt concentration.

**Figure 4:**
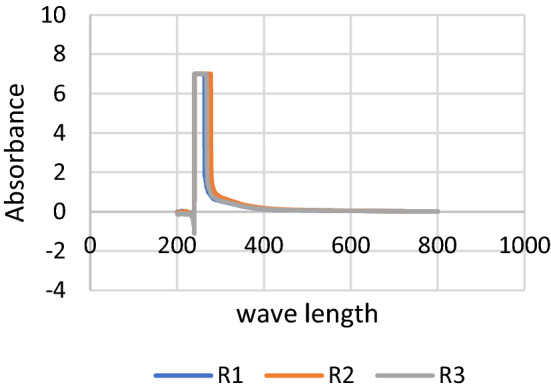
UV–Vis spectrum showing the formation of the nano iron characteristic beak in replicated samples.

Upon addition of the fungal culture filtrate, the reaction mixture showed a color change from clear pale yellow to blackish brown which represents an initial indication for the reduction of Fe^+2^ ions by the culture filtrate and the production of iron nanoparticles, Fig. 3. Moreover, UV–Vis spectrum showed the formation an absorption beak at the triplicated samples at 250–268 nm, Fig. 4.

### Monitoring of iron nanoparticles biosynthesis over different time intervals

**Figure 5:**
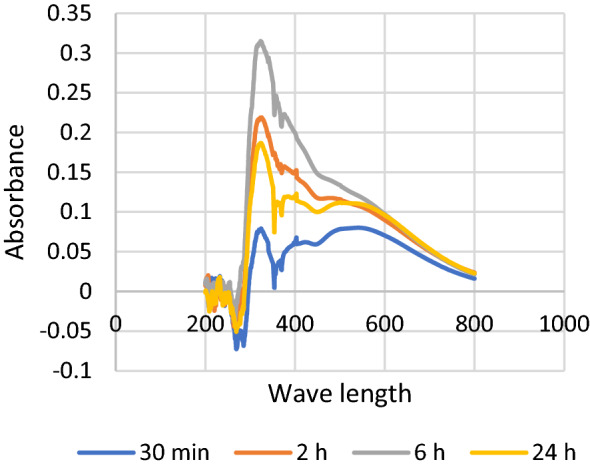
UV–Vis spectrum for tracking nano iron synthesis over different time intervals.

Monitoring of iron nanoparticles’ synthesis over different time intervals showed that, the beak of iron nanoparticles has been detected just after 30 min from the beginning of the reaction and the absorption value increased with time to reach its best reading after 6 h. No shift has been observed in beak position with time (all nearly at 330 nm), Fig. 5.

### Characterization of synthesized iron nanoparticles

#### Dynamic light scattering (DLS) and transmission electron microscopy (TEM)

**Figure 6:**
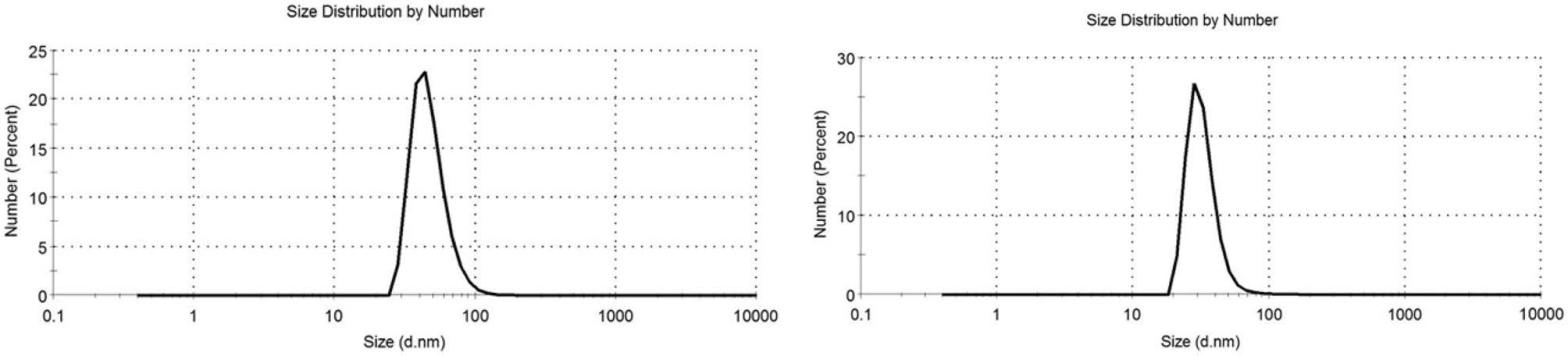
DLS measures showing size distribution of iron nanoparticles synthesized by *Aspergillus flavipes* MN956655.1.

**Figure 7:**
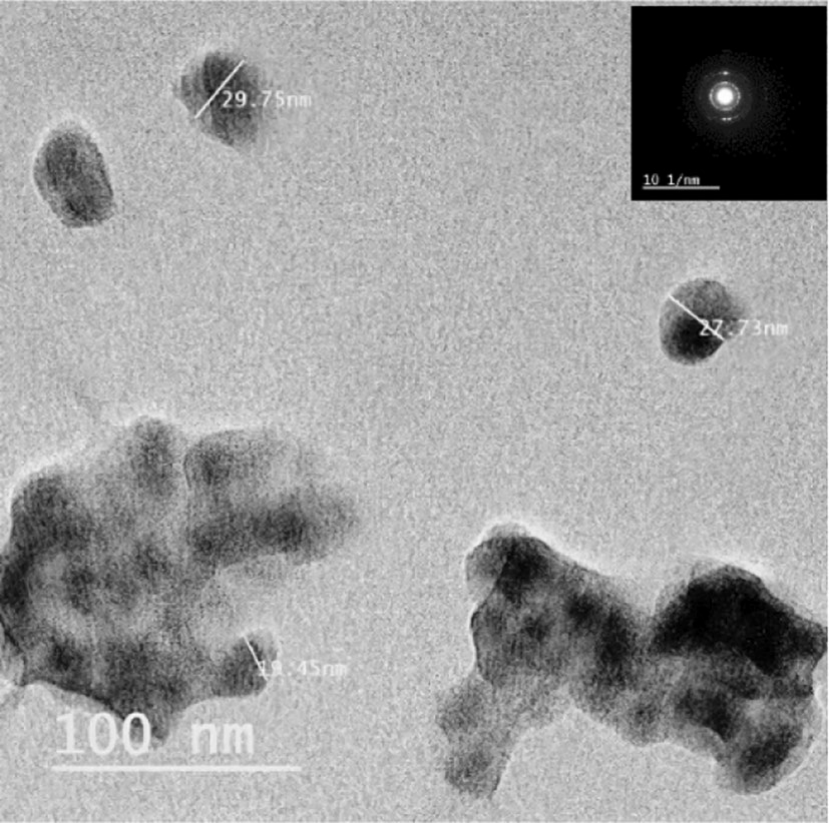
TEM image of *Aspergillus flavipes* MN956655.1 mediated iron nanoparticles showing the electron diffraction pattern on the top.

The Dynamic light scattering analysis showed that the estimated size of the produced iron nanoparticles ranges between 32.7 and 47.6 nm, Fig. 6, while TEM micrographs have recorded their size in range of 19.45–29.75 nm, Fig. 7.

#### Vibrating sample magnetometer (VSM)

**Figure 8:**
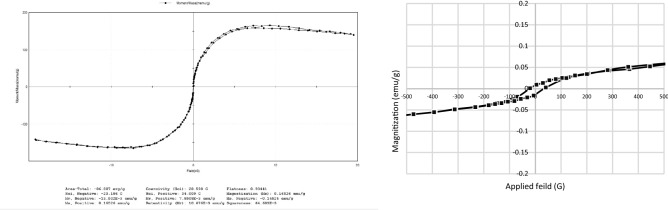
Magnetization curve of the biosynthesized iron nanoparticles showed the formation of a hysteresis loop.

The magnetic behavior of synthesized iron nanoparticles was assessed by Vibrating Sample Magnetometer (VSM). The obtained magnetization curve showed the formation of a hysteresis loop, Fig. 8. The estimated coercivity value (Hc) was 28.598 G (2.8 KA/m). The reported saturation magnetization value of the synthesized particles was 165.2 memu/g.

#### Fourier-transform infrared (FT-IR) spectroscopy

**Figure 9:**
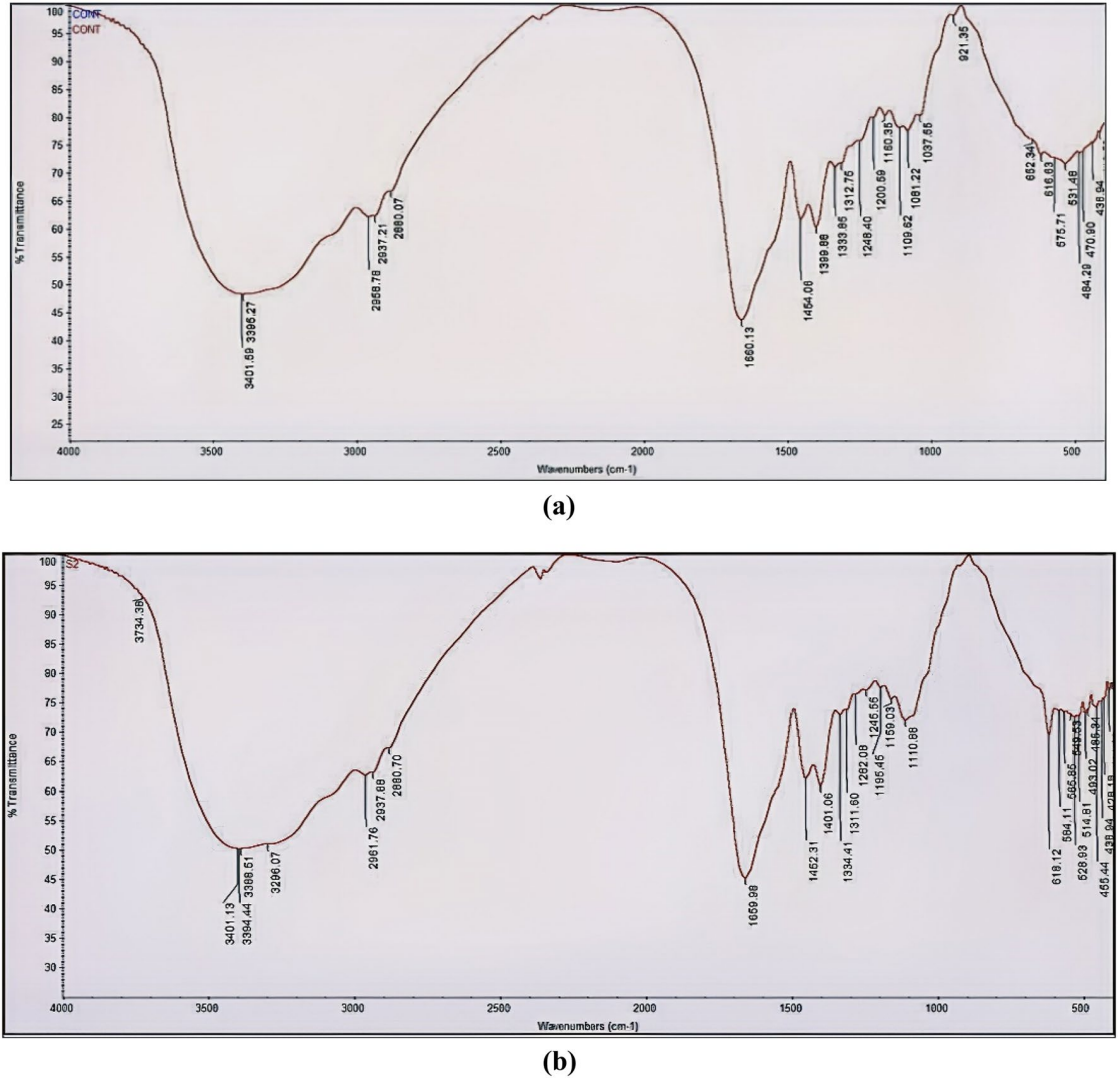
Fourier transform infrared (FTIR) spectra of (**a**) *Aspergillus flavipes* MN956655.1culture filtrate, (**b**) iron nanoparticles synthesized by *Aspergillus flavipes* MN956655.1.

FT-IR spectra of *Aspergillus flavipes* MN956655.1 culture filtrate and iron nanoparticles synthesized by it, Fig. 9, showed common bands at 3401, ~ 2960, 2937, 2880, ~ 1452, ~ 1400, 1660, ~ 1159, ~ 1109, 1200, ~ 1334 and ~ 1311 cm^−1^. On the other hand, FT-IR spectrum of biosynthesized iron nanoparticles showed the appearance of unique beaks in 400–600 cm^−1^ range and a further unique beak at 3734 cm^−1^.

### Optimization of some production parameters using response surface methodology

ANOVA analysis of the employed Box-Behnken model showed that the model *p* value was 0.0084, the model *F*-value was 10.98 and the Lack of Fit was not significant (The “Lack of Fit *F*-value” was 0.43), Table 3. Value of the model Correlation coefficient (*R*^2^) equals 0.95, while the adjusted *R*^*2*^ value equals 0.87, Table 4. According to the statistical analysis, B, BC, A^2^ and C^2^ are significant model terms where P < 0.05. The second-order polynomial equation estimated by the model regression analysis was as following:

$$ {\text{Particle}}\;{\text{size}} = 137 + 9.63{\text{A}} - 126.13{\text{B}} - 21{\text{C}} + 11.5{\text{AB}} - 35.25{\text{AC}} + 107.75{\text{BC}} + 138.75{\text{A}}^{2} + 18.25{\text{B}}^{2} + 117.5{\text{C}}^{2} $$

**Table 3:** Results of ANOVA statistical analysis of tested factors’ effect on size of the produced iron nanoparticles, employing Box-Behnken design.

Variable	Effect (coefficient)	Standard error	*P* value	Significance
A	9.63	19.38	0.6406	Non-Significant
B	− 126.13	19.38	0.0013	Significant
C	− 21.00	19.38	0.3281	Non-significant
AB	11.50	27.41	0.6923	Non-significant
AC	− 35.25	27.41	0.2548	Non-significant
BC	107.75	27.41	0.0111	Significant
A^2^	138.75	28.53	0.0046	Significant
B^2^	18.25	28.53	0.5506	Non-significant
C^2^	117.50	28.53	0.0092	Significant
Model	137.00	31.66	0.0084	Significant

A = Salt conc. “FeSO_4_.7H_2_O”, B = Culture filtrate volume, C = pH.

**Table 4:** ANOVA regression statistics for Box-Behnken design.

Model *R*^2^	0.95
Model adjusted *R*^2^	0.87

where A, B and C are the coded factors of salt concentration, culture filtrate volume and pH respectively. The main effect of these factors and the interaction among them are visualized by Pareto chart, Fig. 10, the perturbation and the three-dimensional response plots. Perturbation plot, Fig. 11, shows the main effect of tested factors on the particle size. It has been observed that the volume of added culture filtrate has the major effect, where the increase in the added volume leads to a reduction in size of the produced iron nanoparticles.

**Figure 10:**
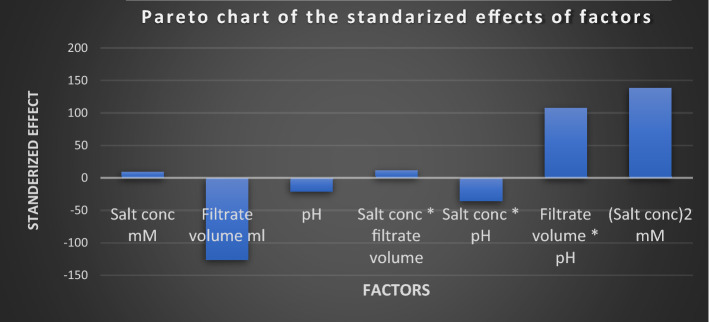
Pareto chart showing the effect of tested factors on particle size of the produced iron nanoparticles according to Box-Behnken design analysis.

**Figure 11:**
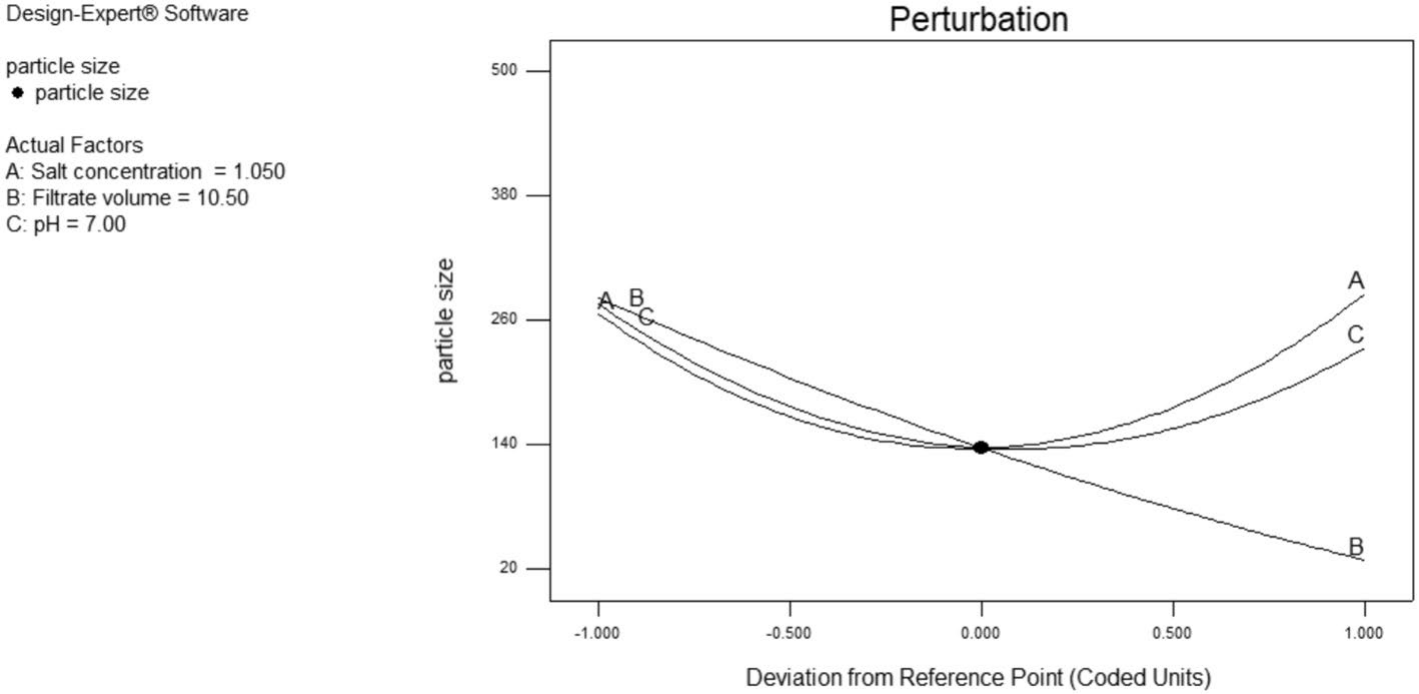
Perturbation plot showing the main effect of A = Salt conc., B = Culture filtrate volume, C = pH on size of produced iron nano particles.

**Figure 12:**
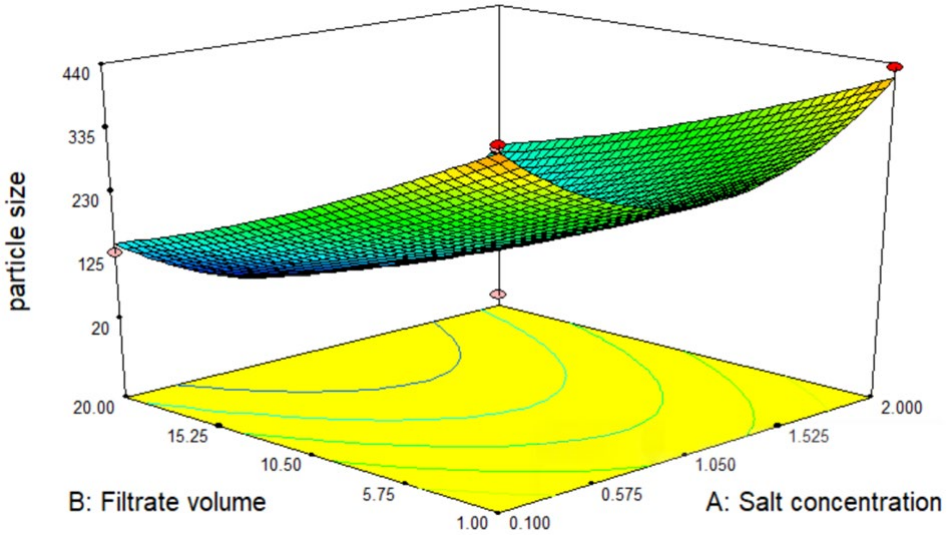
3-D. Response surface plot showing the interaction between the filtrate volume and salt conc. and its effect on the produced particle size.

**Figure 13:**
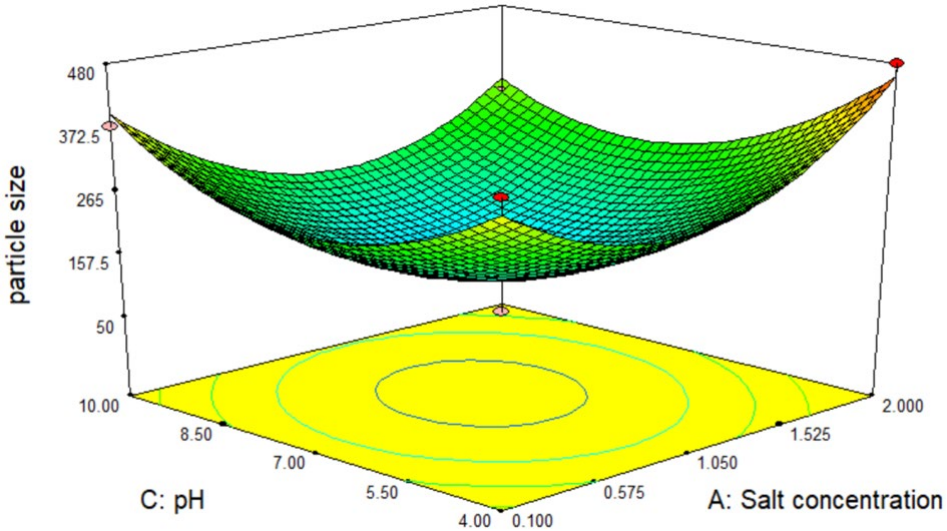
3-D. Response surface plot showing the interaction between the pH and salt conc. and its effect on the produced particle size.

**Figure 14:**
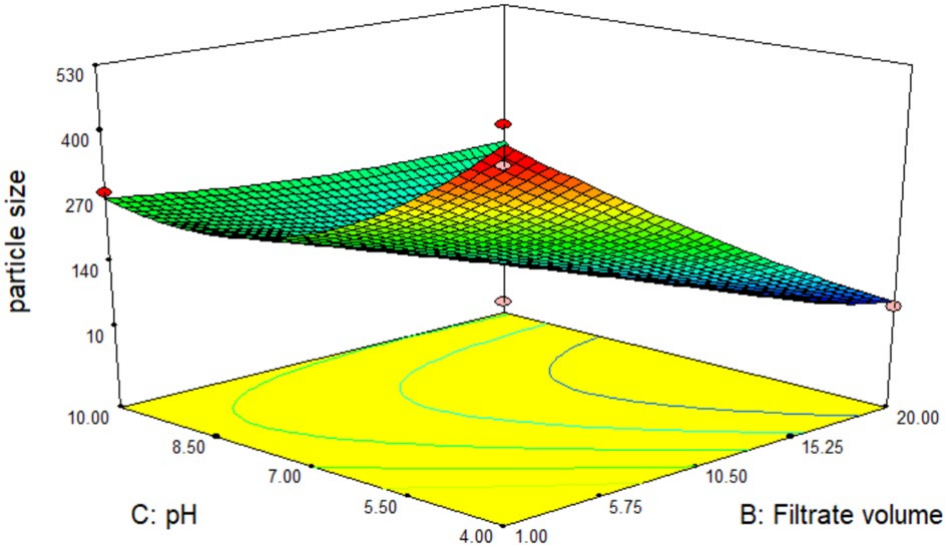
3-D. Response surface plot showing the interaction between the pH and filtrate volume and its effect on the produced particle size.

**Figure 15:**
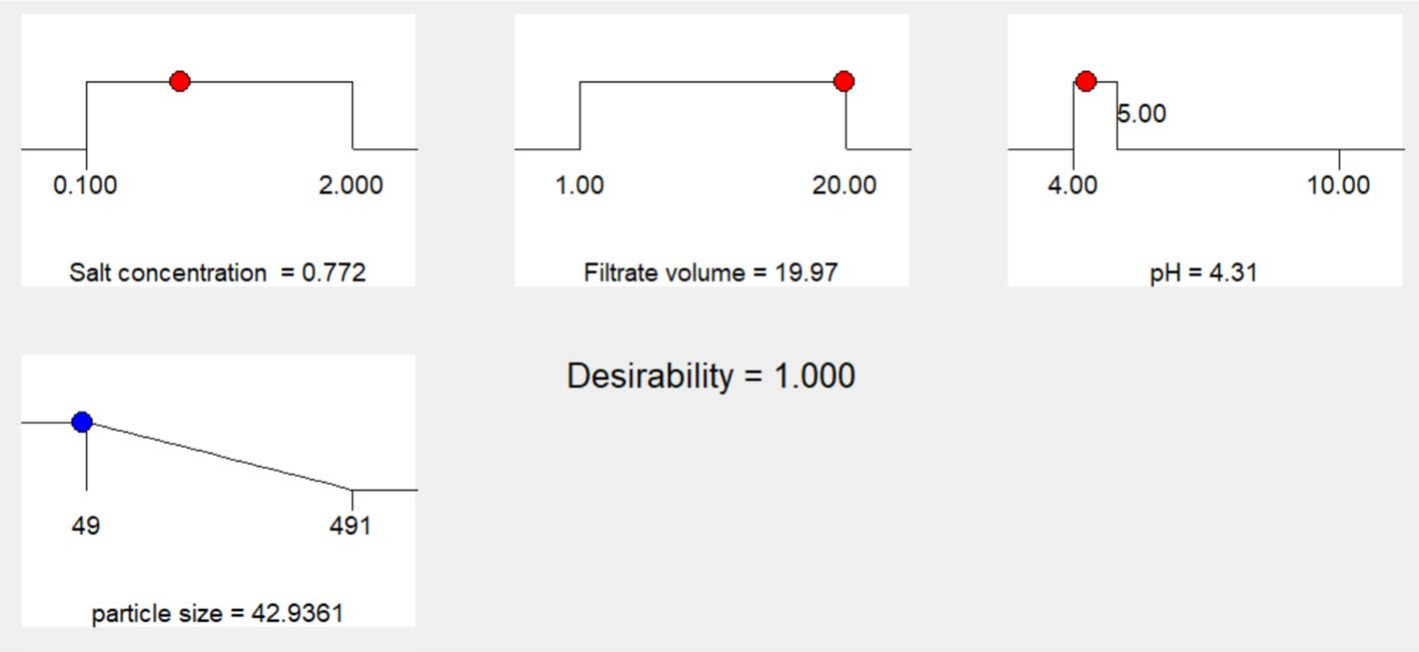
A predicted solution estimated by Box-Behnken design numerical optimization that used in validation of model to yield the smallest particle size.

The 3D response plots, Figs. 12, 13 and 14, were displayed by using two factors and keeping the third at the zero coded level (its intermediate value) to illustrate both the main and the interactive behavior of tested factors. They revealed that there is a significant mutual interaction between the volume of added culture filtrate and the pH value. The smallest particle size which can be obtained was calculated by the aid of Design Expert’s numerical optimization tool which predicts to be 42.9 nm when salt concentration, volume of added culture filtrate and pH equal 0.772 mM, 19.97 ml and 4.31 respectively, Fig. 15.

#### Validation of model

The previous predicted solution for yielding the best particle size was used as a check point for model validation by testing the approximated values of its levels experimentally and comparing the actually obtained result with the predicted one. It has been found that the produced iron nanoparticles have recorded a particle size of 41.5 nm, giving a deviation of 3.3% from the model predicted value, Table 5.

**Table 5:** Model validation results (predicted and actual values for enhancing the main response).

Method	Variable level			Particle size (nm)	Deviation
	Salt*^1^ conc. (mM)	Filtrate*^2^ volume (ml)	pH		
Design Expert’s model prediction	0.772	19.97	4.31	42.9	3.3%
Experimental value (tested/Actual)	0.77	20	4.14	41.5	

### Assessment of production process before and after optimization study

Characters and amount of produced iron nanoparticles have been checked before and after applying the optimized conditions, Table 6, to evaluate the efficiency of the performed optimization study.

**Table 6:** Summary of the optimal levels estimated by Box-Behnken design.

Factor	Standard level	Estimated Optimal level
FeSo_4_.7 H_2_O conc	0.1 mM	0.77 mM
Added filtrate volume	10 ml	20 ml
pH	5.7	4.3

Concerning the particle size, nanoparticles have recorded a size of 48.2 ± 8.9 nm before applying the optimized conditions and a size of 41.5 ± 7.1 nm after applying the optimized levels (*t*-test *P*-Value = 0.308).

**Figure 16:**
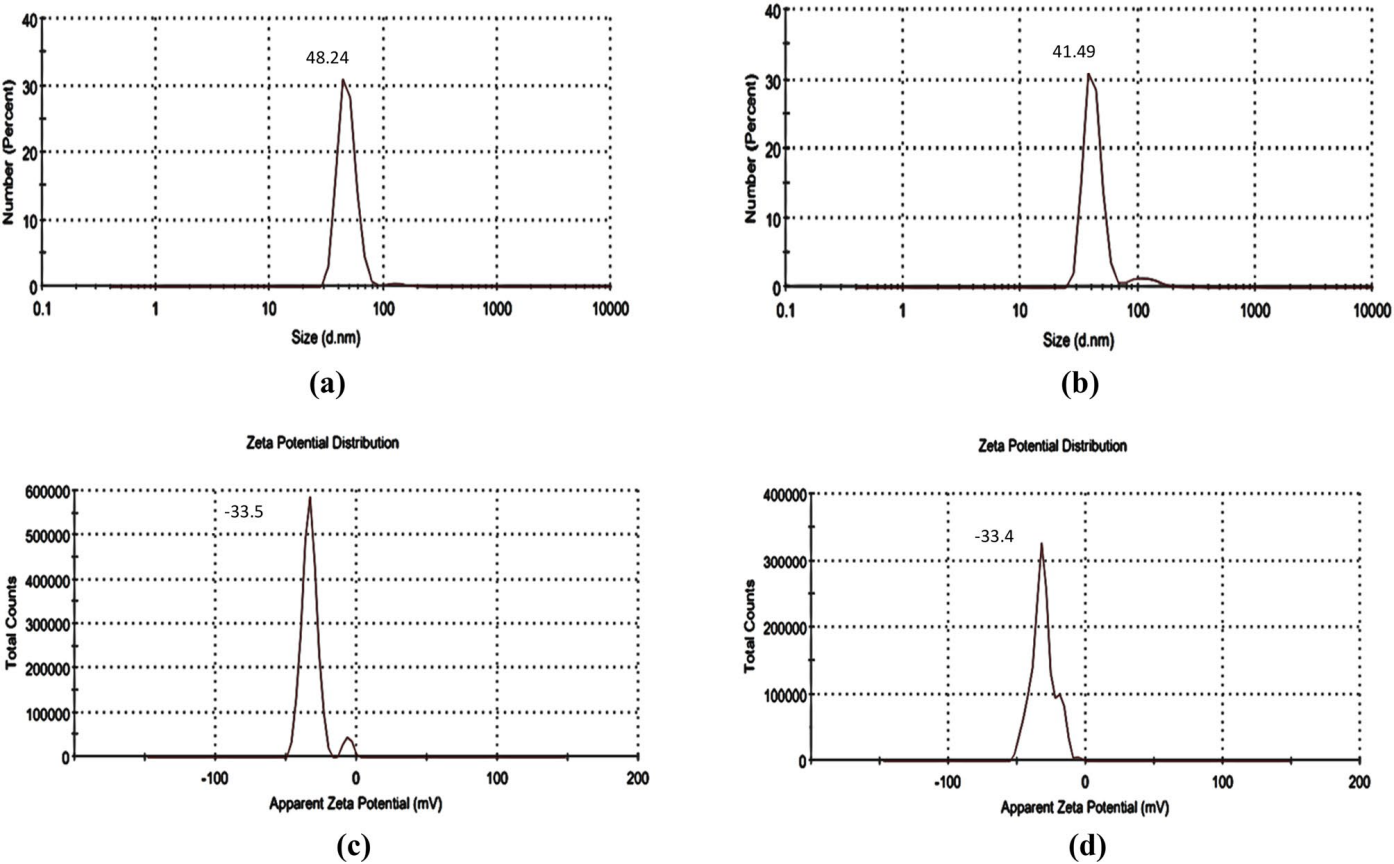
Size (**a**) & surface charge (**c**) of synthesized iron nanoparticles before employing the optimized conditions. Size (**b**) & surface charge (**d**) of synthesized iron nanoparticles after employing the optimized conditions.

Similar response has been observed for surface charge, where zeta potential analysis revealed that the particles have a charge of − 33.5 ± 5.3 and − 33.4 ± 6.5 before and after applying the optimization levels respectively (*t*-test *P*-Value = 0.496), Fig. 16.

**Figure 17:**
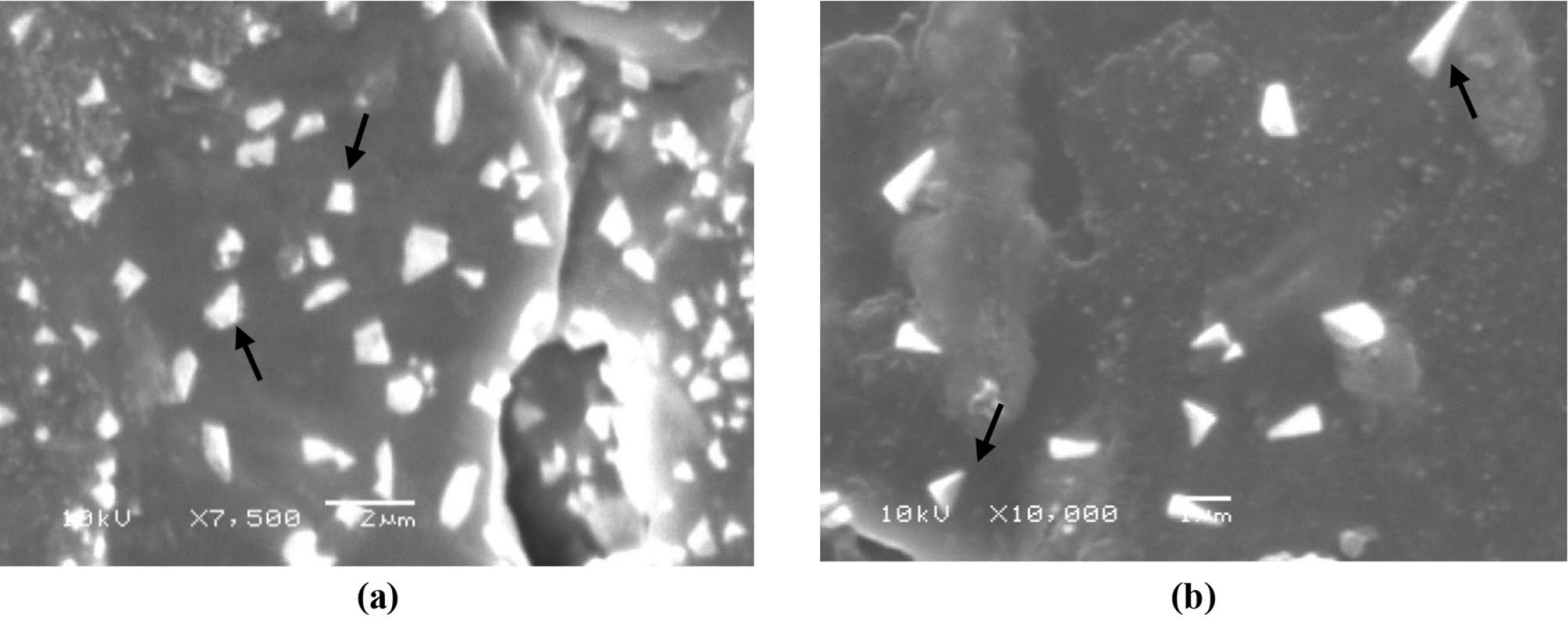
SEM micrographs showing morphology of biosynthesized iron nano particles before (**a**) and after (**b**) optimization study.

Regarding the morphology of the produced iron nanoparticles, SEM results showed that the truncated tetrahedron shape is the most recorder shape among the nanoparticles before optimization study and the tetrahedron shape is the common shape after applying the optimized conditions, Fig. 17.

**Figure 18:**
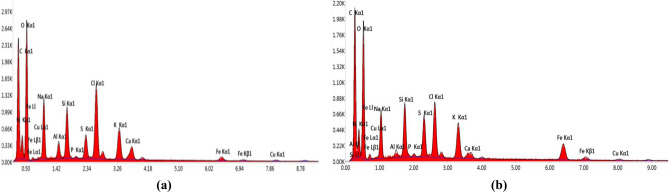
EDX Spectra showing iron percentage in prepared samples before (**a**) and after (**b**) employing optimization process.

Checking the quantity of iron in the prepared nanoparticles before and after employing the optimized conditions has been performed using EDX analysis. EDX spectra, Fig. 18, revealed the presence of absorbance peaks at approximately 1 and 7 keV which are correlated to iron nanocrystal. Other major beaks have been reported for C, N, O and S. Iron percentage in the prepared nanoparticles was 0.97% and 3.87% before and after applying the optimized levels respectively, Table 7.

**Table 7:** Elemental percentage in prepared samples before and after optimization study as estimated by EDX.

Element	C	O	N	Na	K	S	Fe
% After optimization	31.89	32.78	12.61	5.09	3.09	1.48	3.87
% Before optimization	30.65	35.1	11.18	6.54	2.99	2.46	0.97

Concerning the yield, the amount of the produced iron nanoparticles was 4.2 ± 1.6 mg/100 ml before applying the optimized conditions and that amount has increased to 19 ± 2 mg/100 ml after the optimization process (*t*-test *P*-Value = 0.0143).

**Figure 19:**
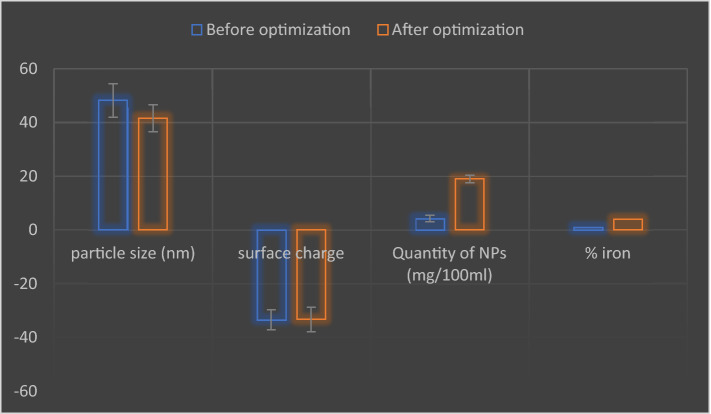
Effect of optimization process on characters and quantity of produced iron nanoparticles.

Figure 19 summarizes the variability in particle size, surface charge, amount of iron nanoparticles and the variability in their iron percent before and after employing the optimization process.

### Effectiveness of the produced magnetic iron nanoparticles as dye decolorizing and Antifungal agent

#### Dye decolorization

**Figure 20:**
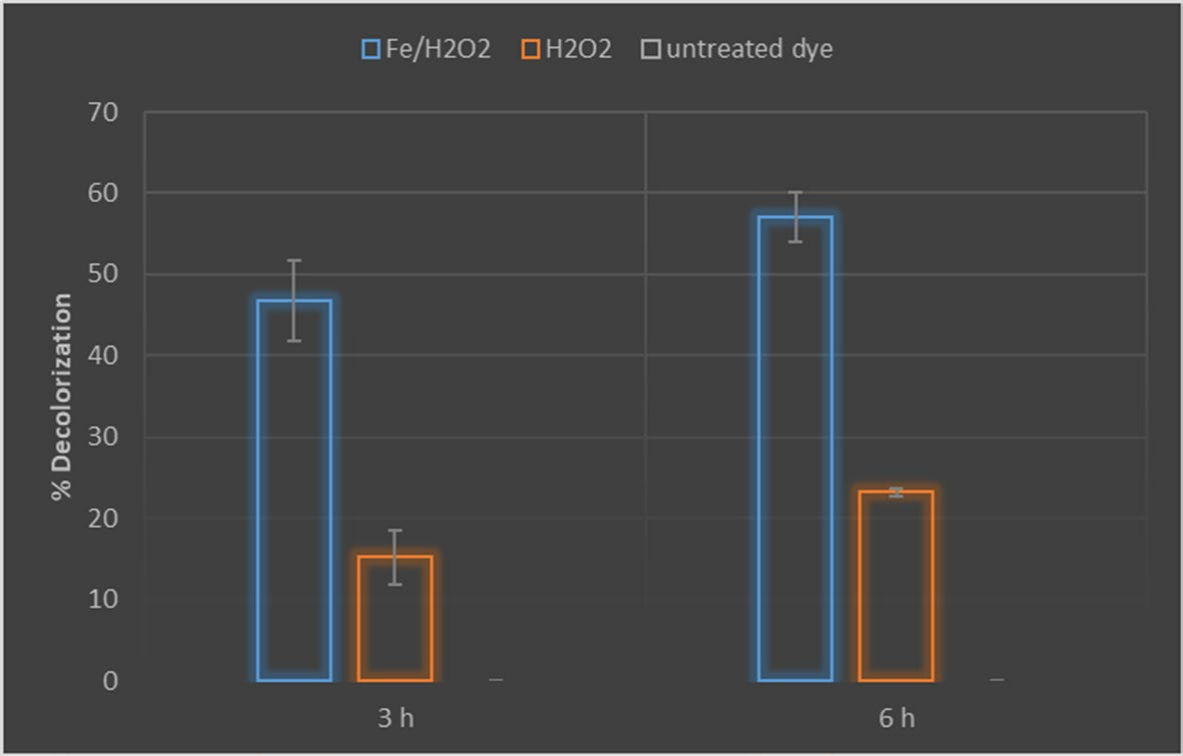
Methylene blue decolorization ability of biosynthesized iron nanoparticles.

Efficacy of the biosynthesized iron nanoparticles in methylene blue decolorization was evaluated in the presence of H_2_O_2_. The obtained results showed that, there is an obvious decrease in absorbance of methylene blue solution treated with iron nanoparticles in comparison to solution treated only with H_2_O_2_ which reflects a decolorization ability of the tested nanoparticles. It has been found that, decolorization percentage reaches 57 ± 4.3% after 6 h in case of iron nanoparticles, but solution treated only with H_2_O_2_ has recorded 23.4 ± 0.7 decolorization percentage. Methylene blue solution without H_2_O_2_ or iron nanoparticles treatments showed zero decolorization percentage after the tested periods, Fig. 20.

#### Antifungal activity

Testing of antifungal activity showed that the use of iron nanoparticles with 50 ppm concentration has exerted a detectable inhibition especially against *Alternaria solani* (18.6 ± 0.8 inhibition percent), Table 8.

**Table 8:** Antifungal activity of biosynthesized iron nanoparticles.

Tested concentration	% Inhibition			
	*F. solani*	*F. semitectum*	*A. solani*	*S. rolfsii*
5 ppm	0.7 ± 0.7	0.7 ± 0.7	4.3 ± 0	0 ± 0
50 ppm	2.7 ± 1.1	12.2 ± 1.1	18.6 ± 0.8	10.4 ± 2.2

## Discussion

In this study, a new *Aspergillus flavipes* isolate has been obtained from soil sediment of *Avicennia marina* (Forssk.) Vierh. growing on Red Sea coast at Ras Mohammad—South Sinai—Egypt. It has been identified according to both its ITS4 sequence analysis and its growth, macroscopic and microscopic characters. Its ITS4 sequence was submitted in GenBank as *Aspergillus flavipes* isolate RMMB and released in GenBank under the accession number of MN956655.1. Production of iron nanoparticles by this isolate has been detected upon the addition of its culture filtrate to the FeSo_4_.7 H_2_O solution, where a color change has been observed from the clear pale yellow to blackish brown which represents an indication for the reduction of Fe^+2^ ions to their zero state. Formation of iron nanoparticles has also been tracked by scanning UV–Vis spectrum of prepared samples. UV–Vis spectrum showed the formation of an absorption beak at 250–268 nm which agree with the optical properties of Surface Plasmon Resonance of the iron nanoparticles and fits the described maximum absorption range characteristic for iron nanoparticles (250–350 nm)^4^. Tracking of the iron nanoparticles production over different time intervals using UV–Vis spectroscopy showed the formation of its characteristic beak just after 30 min. after initiating the reaction; and the position of such beak didn’t shift over the tested time intervals, which can indicate a stabilization in particle size for the formed iron nanoparticles over the tested periods.

The most important parameter that must be considered in synthesis of nanoparticles is their size range where it is significant in determining the effectiveness of such particles in the different application fields^31^. Thus, size of the produced iron nanoparticles has been estimated using dynamic light scattering (DLS) technique and transmission electron spectroscopy (TEM). Dynamic light scattering results has recorded the particle size in the range of 32.7—47.6 nm; and the TEM micrographs have estimated the size in the range of 19.45—29.75 nm. That recorded variability between the DLS and TEM measures is within the accepted range which can be explained by the presence of the nanoparticles during the DLS analysis as a suspended particles in a solution. Hence, they may be surrounded by other ions which can interfere with their exact size^32^. Xiao et al. utilized tea extract in green synthesis of iron nanoparticles but the synthesized particles have recorded size range of 75–100 nm^20^. Also, iron nanoparticles synthesized by endophytic *Penicillium oxalicum* have showed a relatively large size (140–170 nm)^19^. Thus, utilizing *Aspergillus flavipes* isolate RMMB in iron nanoparticles represents an enhanced biosynthesis method in terms of the particle size.

Concerning the magnetic behavior of the biosynthesized iron nanoparticles, it has been observed that; their magnetization curve showed the formation of a hysteresis loop which indicates a ferromagnetic behavior for the synthesized iron nanoparticles. Moreover, the estimated coercivity value (Hc) was 28.598 G (2.8 KA/m). The magnetic materials with coercivity less than 1 kA/m are considered as magnetically soft and the materials with coercivity higher than 10 kA/m are considered as hard magnetic materials^33^. The estimated coercivity value fits the range in between soft and hard ferromagnetic materials which reflects the semihard ferromagnetic behavior of synthesized iron nanoparticles. The reported saturation magnetization value of the synthesized particles was 165.2 memu/g. This value is higher than the magnetization value reported by Kheshtzar et al. for zero-valent iron nanoparticles synthesized by extracts of green tea leaves which was around 80 memu/g^34^. Also, that recorded value is better than the magnetization value reported for iron oxide nanoparticles coated with green tea polyphenols and those synthesized by tannin extract from *Acacia mearnsii* where their values were 61 memu/g^35^ and 3.2 memu/g^36^ respectively. These reduced values were explained by the diamagnetic effect of capping biological materials and amorphous state of the prepared iron nanoparticles^34^. Thus, the obtained results reveal an enhanced magnetic property for the biosynthesized iron nanoparticles compared to other green synthesized ones giving them more suitable characteristics for the remediation applications as the magnetic manipulation is required.

FT-IR spectroscopy was employed to identify and characterize the functional groups associated with the biosynthesis and stabilization of iron nanoparticles. Monitoring of such functional groups can be used in interpretation of the chemical nature of biomolecules that may be involved in synthesis and capping of biosynthesized nanoparticles. FT-IR spectra of *Aspergillus flavipes* MN956655.1 culture filtrate and iron nanoparticles synthesized by it showed a significant common band at 3401 cm^−1^ which corresponds to bonded O–H stretch^31,37,38^. Also, they revealed other common bands at ~ 2960, 2937, 2880, ~ 1452 and ~ 1400 cm^−1^ that are related to C–H and C–H_2_ stretching^4,31,37–39^. Another common band in 1660 cm^−1^ is observed which reflects aromatic combinations and C=C aromatic ring stretching vibration^31,34,37,38^. On the other hand, presence of bands in ~ 1159 and ~ 1109 cm^−1^ represents possible indication for C–O stretch for tertiary and secondary alcohol respectively^37,38^. Moreover, a band in 1200 cm^−1^ is detected which corresponds to phenolic C–H stretch^34,37,38^. All previous signals reveal possible involvement of flavonoids, phenolic^4^ and alcoholic compounds in synthesis and capping of iron nanoparticles. In addition to that, common bands have been observed in ~ 1334 and ~ 1311 cm^−1^ which may belong to aromatic C–N stretching^34,37,38^ and that indicates a possible role of the aromatic nitrogenous compounds in synthesis and capping process. FT-IR spectrum of biosynthesized iron nanoparticles showed the appearance of unique beaks in 400–600 cm^−1^ range which correlated to Fe–O stretching vibration mode^31,34,40^ and that confirming the formation of magnetite iron nanoparticles. A further unique beak has been observed at 3734 cm^−1^ which corresponds to nonbonded hydroxyl group O–H^37,38^ stretch that may be due to residual water which surrounds the nanoparticles.

An optimization study has been performed employing experimental design method to reveal how to employ the production process under the most efficient conditions to enhance the iron nanoparticles’ production process. Experimental design methods represent a competitive and more effective methods for optimization process than the traditional method ‘one factor at time’ as the experimental design methods not only illustrate the effects of tested factors and optimize their levels but also can estimate the interaction between them^41,42^. As illustrated from the previous studies; pH, the volume of added culture filtrate and the used metallic salt concentration are among the most important factors affecting the nanoparticle synthesis process. Hence these three factors have been selected for the optimization study using experimental design method. ANOVA analysis of the employed Box-Behnken model showed that, the model is significant where the model *p* value was 0.0084. Also, it has been found that the value of the model Correlation coefficient (*R*^2^) equals 0.95 and the adjusted *R*^2^ value equals 0.87, which reflects a good correlation and supports the significance of the model. Concerning the main effect of tested factors, the volume of added culture filtrate has recorded the major effect, where the increase in the added volume leads to a reduction in size of the produced iron nanoparticles. Visualization of the interactive behavior of tested factors using the 3D response plots revealed the presence of a significant mutual interaction between the volume of added culture filtrate and the pH value. The model has predicted that, the smallest particle size which can be obtained is 42.9 nm when salt concentration, volume of added culture filtrate and pH equal 0.772 mM, 19.97 ml and 4.31 respectively. This prediction was tested experimentally for model validation, and it has been found that; employing the production process under the prementioned levels yields iron nanoparticles with a size of 41.5 nm giving a deviation of 3.3% from the model predicted value and that represents an acceptable error range which recommends the validation of the employed model. Similar results have been reported by Kheshtzar et al., where they have recorded a significant role for the volume of green tea extract used in biosynthesis of iron nanoparticles after employing an optimization study using a response surface methodology design^34^. Also, Singh et al. have optimized biosynthesis of iron oxide nanoparticles by leaf extract of *Coriandrum sativum* L. (cilantro) utilizing response surface methodology and they have estimated that, the volume of added leaf extract is a significant factor in synthesis process. However, their optimization study failed to reduce the size of the produce nanoparticle below 161 nm^43^.

The efficiency of the performed optimization study was evaluated by comparing the size, shape, amount of the produced iron nanoparticles before and after employing the production process under the optimized conditions. conditions. Concerning the particle size, applying the optimized levels has led to a slight reduction in particle size. However, this size reduction was found to be statistically non-significant (*t*-test *P*-Value = 0.308). A similar response has been observed for surface charge. The biosynthesized iron nanoparticles have recorded a negative surface charge which can support their application in bioremediation process where they can attract and absorb positively cationic dyes. Moreover, the estimated value of surface charge (− 33.4 ± 6.5) can be considered high enough to maintain a reasonable stability for the prepared nano solution over storage. This reported value is better than the surface charge of iron oxide nanoparticles synthesized by *Coriandrum sativum* leave extract even after employing the optimization process where they have recorded only a potential of − 19.5^43^.

Regarding the morphology of the produced iron nanoparticles, SEM results showed a clear variability in shape and topographic characters before and after applying the optimized conditions. The most common shape that has been observed among the nanoparticles before optimization study is the truncated tetrahedron while the most recorded shape after applying the optimized conditions is the tetrahedron which can be correlated to the increased salt concentration used in the production process. Similar behavior was reported by Zheng et al. during the preparation of gold nanoparticles, where the increase in gold precursor amount stimulated the formation of tetrahedron particles rather than the truncated tetrahedron shape^44^.

EDX analysis was performed to evaluate the elemental composition of prepared samples and check the quantity of iron in them before and after employing the optimized conditions. EDX spectra revealed the presence of absorbance peaks associated with surface plasmon resonance of iron nanocrystal at approximately 1 and 7 keV^6,45^. In addition to that, an enhanced iron percent has been obviously detected after applying the optimized levels where it has been increased from 0.97% to 3.87% (increased fourfold). Other major beaks have been reported for C, N and O that may be correlated to the biomolecules that involved in capping of iron nanoparticles, as discussed in results of FTIR analysis, while S may be derived from FeSo_4_ salt used as a precursor in synthesis process.

Regarding to the yield, it has been found that the amount of produced iron nanoparticles was significantly increased after employing the production process with the optimized conditions, where it increased from 4.2 ± 1.6 mg/100 ml to 19 ± 2 mg/100 ml (*t*-test *P*-Value = 0.0143; fourfold and half increase).

Hence it can be concluded that; the performed optimization process has greatly affected the morphology of the produced iron nanoparticles where it stimulated the formation of particles in tetrahedron shape without increasing the particle size or changing the value of their surface charge. Moreover, the optimized conditions have increased the yield of the produced iron nanoparticles nearly by fourfold.

Effectiveness of the produced magnetic iron nanoparticles in decolorization of methylene blue has been estimated. Use of iron nanomaterials in water treatment processes represent a highly significant technique compared to the other nanomaterials mediated approaches such as adsorption processes (using nano clays and ceramics), use of nanofiltration techniques, use of carbon nanotube and nano-catalyst (for example: titanium dioxide). Iron nano particles show some important characters making them an efficient tool for this purpose as they are less toxic, biodegradable and with high reactivity and large surface area. Additionally, they have a magnetic affinity and dual redox properties on water. Moreover, the precursors used in synthesis of iron nanoparticles are widely available and have low cost^46^. Zero-valent iron nanoparticles employ high adsorption ability, strong reducibility and good catalytic activity; thus, they alone can act as an efficient reducing agent in bioremediation processes. However, their efficiency can be enhanced in presence of oxygen compounds such as hydrogen peroxide, where in this case they produce reactive oxygen species efficiently that play a role in the degradation of organic contaminants^34^. Iron nanoparticles synthesized in this study has recorded good decolorization percent for methylene blue in presence of H_2_O_2_ where they have yielded 57 ± 4.3 decolorization percent after 6 h. Similar results have been recorded for iron oxide nanoparticles which yielded 55.25% degradation for methylene blue^47^ and these recorded decolorization values are better than that recorded for silver nanoparticles synthesized by *cyanobacteria* where they showed only 18% decolorization for methylene blue after 4 h^48^. Although the biosynthesized iron nanoparticles have been tested in this study with low concentration (0.1 mg/ml), they have yielded a good decolorization percent “46.8 ± 4%” for methylene blue after 3 h comparing to the efficiency of zero valent iron nanoparticles biosynthesized by Kheshtzar et al. in decolorization of methyl orange where they have yielded a similar decolorization percent after 2 h but by using higher nanoparticle concentration “1 mg/ml”^34^.

Finally, the antifungal activity of the biosynthesized iron nanoparticles has been estimated against different fungal plant pathogens. It has been found that, these nanoparticles have exerted a detectable inhibition against *Alternaria solani* (18.6 ± 0.8 inhibition percent) when tested with only 50 ppm concentration. Ali et al. have recorded a relatively similar inhibition percent (23.9 ± 0.0) against *A. alternata* when it was treated with higher nano iron concentration (100 ppm/ “0.1 mg/ml”). On the other hand, iron oxide nanoparticles showed lower antifungal behavior against *Alternaria alternata* when tested by the same concentration^49^. Also, Parveen et al. have recorded a lower antifungal activity for iron oxide nanoparticles synthesized by tannic acid against *Alternaria alternata* by assessing their inhibition for spore germination, as 100 ppm concentration (0.1 mg/ml) has yielded only 8.59% inhibition for spore germination^50^.

## Conclusion

Magnetic iron nanoparticles have been synthesized by a green method using *Aspergillus flavipes* MN956655. This is the first study carried out on the biosynthesis of magnetic iron nanoparticles by *Aspergillus flavipes*. This investigated biosynthesis method yields magnetic iron nanoparticle with competitive characters compared to magnetic iron nanoparticles synthesized by plant extracts. The produced nanoparticles have recorded a good size range (32.7–47.6 nm) and a surface charge of − 33.5 ± 5.3 which can maintain a reasonable stability for the prepared nano solutions over storage. These particles showed a semihard ferromagnetic behavior and a magnetization value higher than that recorded for many biosynthesized iron oxide and zero valent iron nanoparticles which reflects less diamagnetic effect of capping biomaterials used in this synthesis process. Optimized conditions stimulated the formation of nanoparticles in tetrahedron shape rather than the truncated tetrahedron shape and efficiently increases the quantity of iron in prepared samples and the amount of produced iron nanoparticles to a fourfold. These biosynthesized iron nanoparticles showed good decolorization behavior for methylene blue. These findings can be utilized in solving one of the potential environmental issues as these nanoparticles can be used in decolorizing of dyes in water in a trial to treat industrial effluents and minimize their environmental hazards. Moreover, the produced iron nanoparticles have exerted a detectable antifungal activity against one of the most important plant pathogens “*Alternaria solani*” with only 50 ppm concentration.

## Data Availability

All data generated during this study are included in this published article. Other raw data analyzed during the current study are available from the corresponding author on reasonable request.
